# An Unusual Postoperative Complication of Retrosternal Gastric Pull-Up for Corrosive Esophageal Stricture

**DOI:** 10.7759/cureus.12323

**Published:** 2020-12-27

**Authors:** Utpal Anand, Ramesh Kumar, Rajeev N Priyadarshi, Kunal Parasar, Aaron G John

**Affiliations:** 1 Surgical Gastroenterology, All India Institute of Medical Sciences, Patna, IND; 2 Gastroenterology, All India Institute of Medical Sciences, Patna, IND; 3 Radiology, All India Institute of Medical Sciences, Patna, IND

**Keywords:** gastric pull-up, caustic ingestion injury, esophageal stricture

## Abstract

Retrosternal gastric pull-up with side-to-side esophagogastric anastomosis is the surgery done for corrosive esophageal stricture unresponsive to endoscopic dilatation. This surgery is considered safe in terms of morbidity. Complications due to leak from distal esophageal staple line as a result of partially patent bypassed esophageal lumen have never been reported with this surgery. Herein, we report a case in which a leak from distal esophageal staple line resulted in intraabdominal fluid collection, a life-threatening complication. This complication necessitated a second surgery that involved exclusion of the native esophagus at the neck.

## Introduction

Esophageal stricture due to corrosive injury is common in India. Such strictures are usually managed by endoscopic dilatation. However, the endoscopic approach can fail to achieve adequate dilatation in severe or long strictures. Such strictures are managed by surgical treatment involving retrosternal gastric pull-up with side-to-side esophagogastric anastomosis. Due to the presence of adhesions, micro-perforation or associated mediastinitis, excision of the native esophagus is dangerous and usually not required [1]. Complete obliteration of esophageal lumen due to corrosive prevents accumulation of food material in natural lumen is another rationale behind leaving the native esophagus. Therefore, circumferential mobilization and closure of the esophagus are often not required in the neck preventing the risk of recurrent laryngeal nerve damage. However, food material can accumulate if the lumen is rather partially obliterated, thus building up the intraluminal pressure that can cause disruption of the intraabdominal esophageal staple line, a life-threatening complication. We report this complication in a young patient, which was ultimately managed by the surgical exclusion of esophagus in the neck. 

## Case presentation

A 25-year-old male presented with dysphagia due to accidental ingestion of a corrosive substance leading to esophageal stricture. He had undergone serial endoscopic dilatations of stricture for three months. However, due to persistent stricture and progressive dysphagia, he was referred to the surgical department. His upper gastrointestinal (GI) endoscopy revealed a non-passable stricture 25 cm from the incisors. Barium swallow test revealed a stricture in the thoracic esophagus.

**Figure 1:**
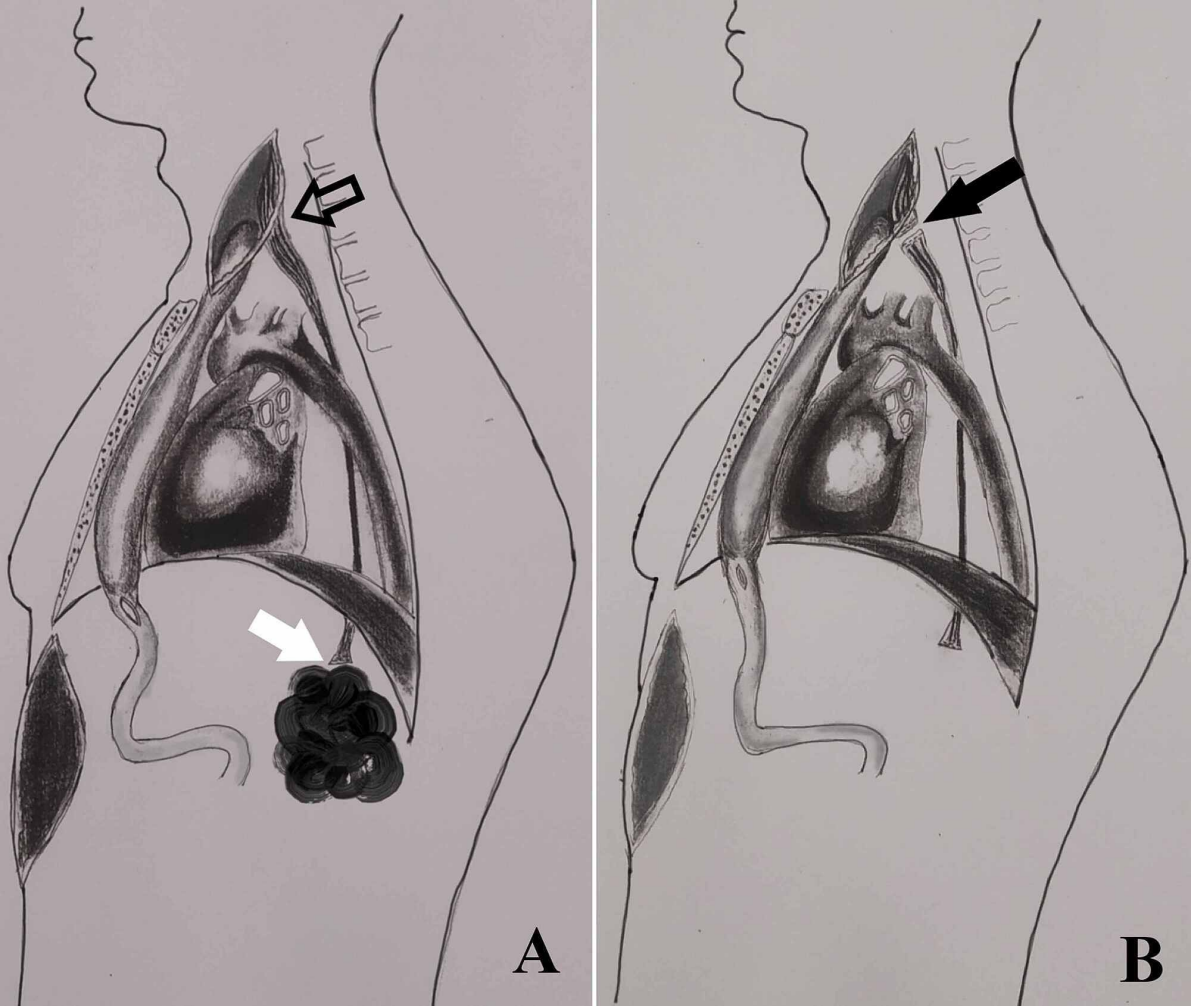
Freehand illustration of the anatomy. (A) Anatomy after the first surgery, retrosternal gastric pull-up with side-to-side esophagogastric anastomosis (black hollow arrow) demonstrating the site of leak (white arrow). (B) Anatomy after the second surgery, bipolar esophageal exclusion demonstrating the site of esophageal stapling (black arrow).

After preoperative evaluation, a retro-sternal gastric pull-up was performed, with the native esophagus being retained in situ and the lower end of the esophagus was stapled in the abdomen. A side-to-side anastomosis of cervical esophagus was performed with a gastric tube in the neck with the presumption of complete obliteration of esophageal lumen (Figure 1A).

Excision of the diseased native esophagus was not attempted. A feeding jejunostomy was performed.

**Figure 2:**
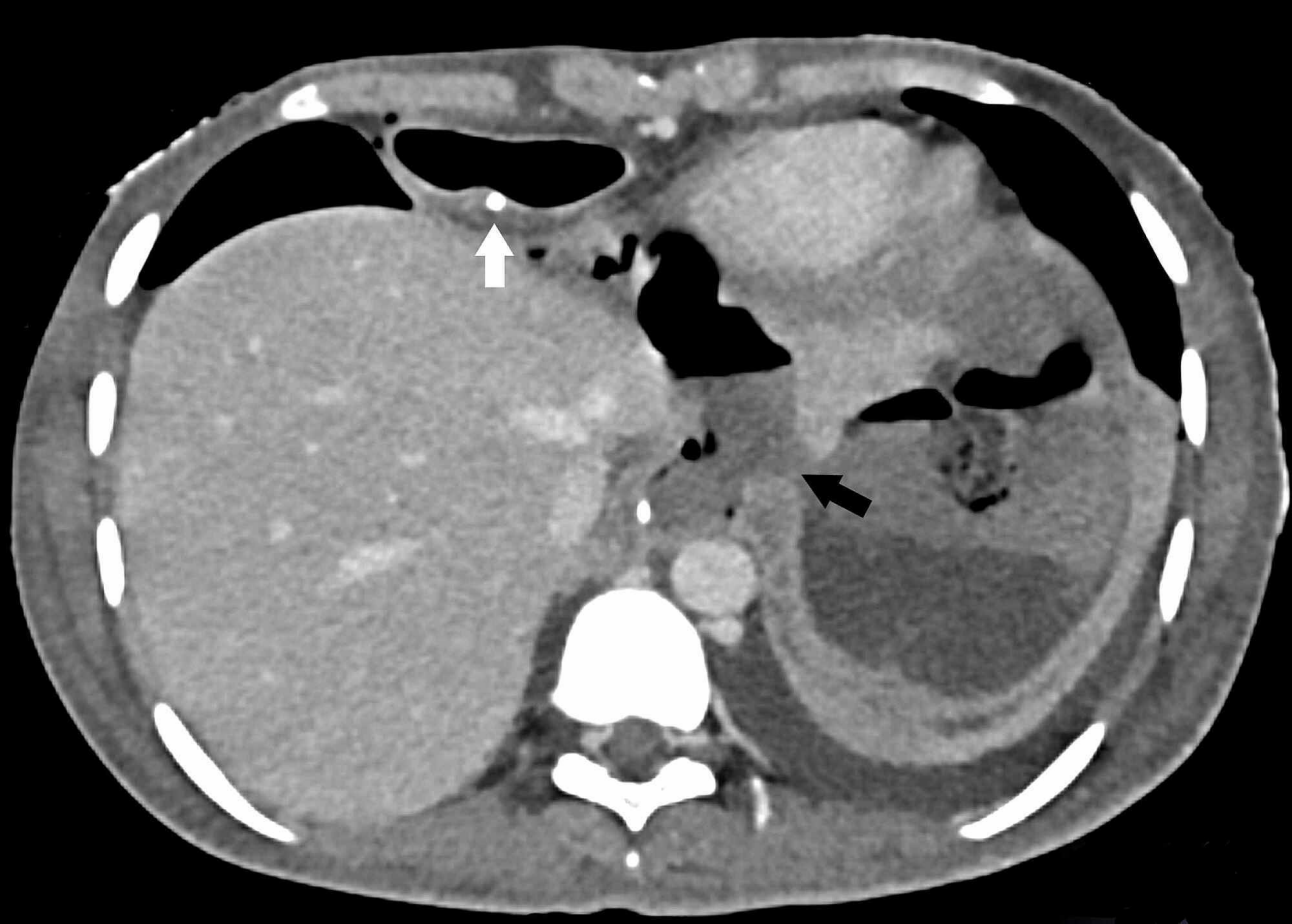
Axial view of abdominal computed tomography with contrast showing the site of leak from the intraabdominal esophageal staple line (black arrow) and the intrabdominal part of the gastric pull-up with intact staple line (white arrow).

On post-operative day 7, the patient was put on a liquid diet after confirming a patent neck anastomosis by oral gastrograffin. The patient tolerated normal diet for two days. On day 9, the patient developed fever, tachypnea and pus discharge from the main abdominal wound. Contrast-enhanced computed tomography (CECT) scan of the chest and abdomen revealed a 3 x 5 cm intraabdominal collection and disruption of intraabdominal esophageal staple line (Figure 2).

**Figure 3:**
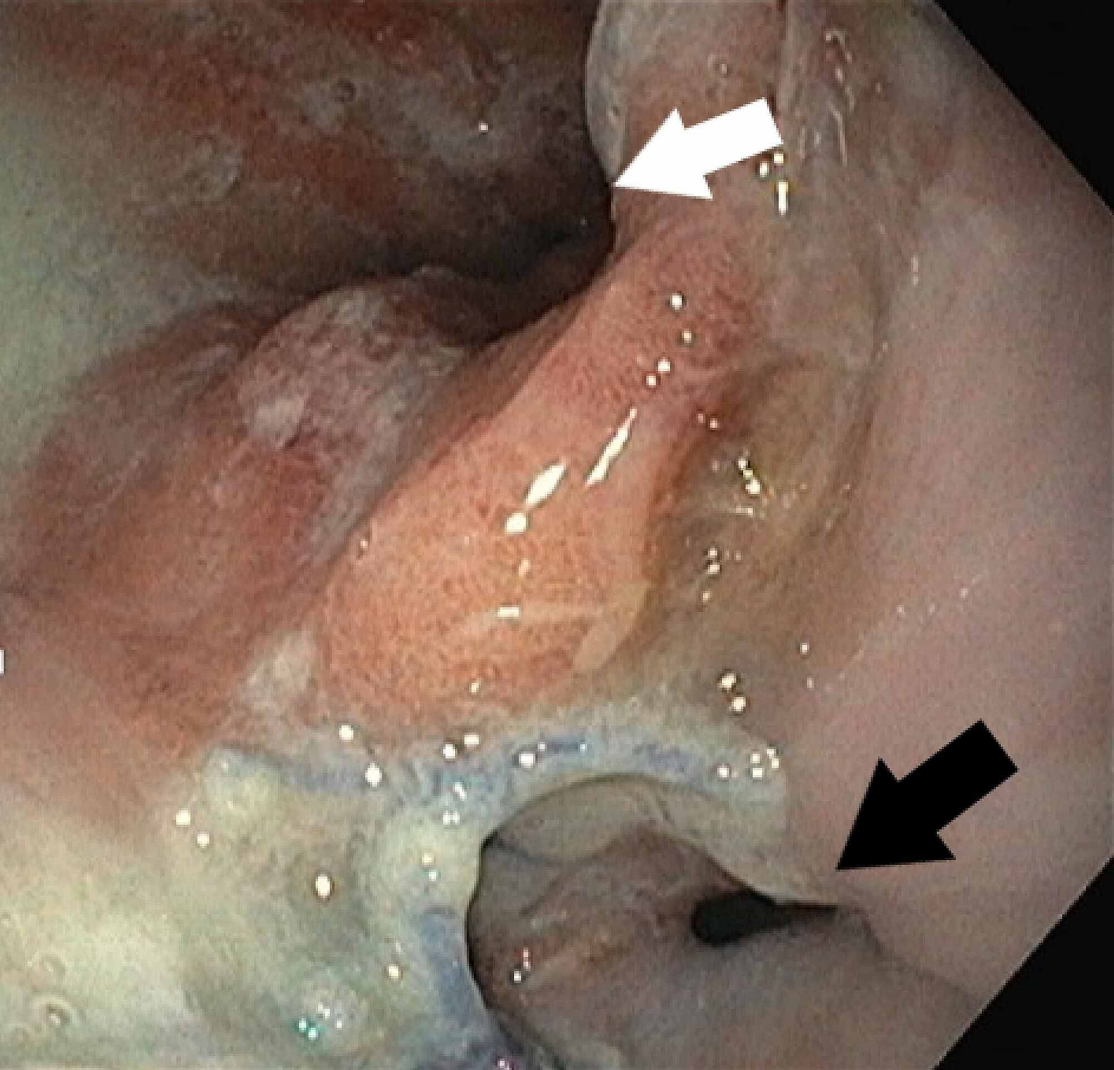
Endoscopic image showing patent narrowed native esophagus (black arrow) and the normal gastroesophageal anastomotic site (white arrow).

A subsequent upper GI endoscopy revealed a normal gastroesophageal anastomotic site and the pulled-up stomach; however, his native esophagus was found leaking into the abdomen (Figure 3).

A persistent leak from the opened stapled esophagus resulted in a large intraabdominal fluid collection. The patient was initially managed with an ultrasound-guided percutaneous drainage and enteral nutrition via feeding jejunostomy. But the catheter drain output continued indicating persistent a leak.

As the general condition of the patient did not improve, a cervical re-exploration and a complete surgical exclusion of the esophagus was performed (Figure 1B). There were significant adhesions in the neck region. In the post-operative period, the patient developed esophagogastric anastomotic leak in the neck, which was managed conservatively by the opening of wound and dressing. Oral feeding was continued with digital compression applied over the wound site. The leak healed within 10 days. On follow-up at three months, the patient was free from dysphagia and had gained adequate weight.

## Discussion

A side-to-side esophagogastrostomy is a standard surgery for corrosive stricture wherein the native esophagus is not resected. Because the native esophageal lumen is usually completely obliterated due to corrosive injury, leak from distal staple line has never been reported [2]. 

This case demonstrates a life-threatening complication due to leak from the intraabdominal esophageal staple line. The leak was due to the passage of food through the incompletely obliterated natural esophageal lumen

Whether to bypass or resect the esophagus in corrosive injury is often a debatable issue [3,4]. The scarred esophagus due to corrosive injury has dense adhesions and its resection is associated with an increased risk of bleeding and damage to trachea and laryngeal nerves [2].

We do not divide the esophagus at the neck as it prevents a blind segment and any secretions from the remnant cicatrized esophagus can drain through the proximal anastomosis avoiding mucocele formation [5]. There are few reports of mucocele formation after bipolar exclusion in corrosive esophageal strictures [1,2,6].

In our case, an incomplete obliteration of the native esophageal lumen resulted in the passage of liquid contents through it and building a high pressure inside a closed lumen. This high pressure might have overwhelmed the holding capacity of stapled esophageal line leading to rupture and leakage of secretions along with food particles free into the abdominal cavity and formation of intrabdominal collection. In order to prevent this complication, a useful alternative can be to drain the lower end of the retained native esophagus into a loop of jejunum to prevent food stasis in thoracic esophageal lumen. Alternatively, when colon is being used as the bypass conduit, the gastroesophageal continuity should be preserved [7,8]. Since the general condition of our patient was prohibitive for a major surgical procedure, we went ahead with the bipolar exclusion of the esophagus by stapling the cervical esophagus and thus disrupting the luminal continuity of the esophagus.

## Conclusions

In conclusion, the importance of this case lies in the fact that the thorough evaluation for complete obliteration of esophageal lumen is required before attempting side-to-side esophagogastric anastomosis in neck; otherwise bipolar exclusion of esophagus is a better option in order to prevent catastrophic complication of intraabdominal esophageal leak due to a partially patent lumen.
